# Association between dietary niacin intake and mortality among US individuals with chronic obstructive pulmonary disease: data from the national health and nutrition examination survey 1999–2018

**DOI:** 10.3389/fnut.2025.1471549

**Published:** 2025-06-09

**Authors:** Chengfeng Fu, Lixia Zhang, Jing Luo, Yingying Zhang

**Affiliations:** ^1^Respiratory and Critical Care Medicine, The Second People’s Hospital of Banan District, Chongqing, China; ^2^Department of Respiratory Medicine, The Second Affiliated Hospital, Chongqing Medical University, Chongqing, China; ^3^Department of Laboratory Medicine, Affiliated Wuxi Fifth Hospital of Jiangnan University, Wuxi, China

**Keywords:** all-cause mortality, cardiovascular disease mortality, chronic obstructive pulmonary disease, dietary niacin intake, national health and nutrition examination survey

## Abstract

**Background and aims:**

Chronic obstructive pulmonary disease (COPD) is a long-lasting condition that significantly hinders respiratory function. Niacin, a crucial nutrient in the diet, is essential for maintaining general health. However, research on the effects of niacin on the mortality risk among individuals with COPD is sparse. Hence, this study aims to investigate the relationship between dietary niacin intake and mortality within this specific cohort of individuals.

**Methods and results:**

A total of 3,674 self-reported COPD individuals from the National Health and Nutrition Examination Survey (NHANES) 1999–2018 were included in this study. The Cox proportional hazards model assessed the association between niacin intake and all-cause/cardiovascular disease (CVD) mortality. Kaplan–Meier curves illustrated survival based on niacin intake. Subgroup and sensitivity analyses were conducted to assess the robustness of the results. During an average follow-up period of 8.3 years, 1,085 all-cause deaths occurred, including 323 CVD-related deaths. A 10 mg/day niacin increase was associated with a 12% lower all-cause and 13% lower CVD mortality risk. Compared to the low dietary niacin intake group, the high intake group had a hazard ratio (HR) of 0.67 (95% CI: 0.56–0.82, *p* < 0.001) for all-cause mortality and 0.53 (95% CI: 0.37–0.77, *p* = 0.001) for CVD mortality. Kaplan–Meier survival curves indicated higher survival rates in the high-niacin group.

**Conclusion:**

A higher dietary intake of niacin was associated with lower all-cause and CVD mortality among individuals with COPD.

## Introduction

Chronic obstructive pulmonary disease (COPD) is a severe chronic respiratory illness characterized by irreversible and persistent airflow limitation in the airways, thus posing a significant global health challenge (1, 2). With a high disability rate and ranking as the third leading cause of death globally, COPD imposes a considerable burden on clinical healthcare systems and public health (3, 4). COPD not only increases overall mortality but is also closely linked to the risk of cardiovascular disease (CVD) death (5). Pathophysiological changes in COPD patients, including systemic inflammation, vascular dysfunction, and hypoxia, accelerate atherosclerosis, thereby increasing the risk of cardiovascular events like myocardial infarction and stroke, and ultimately leading to higher CVD-related mortality (6). COPD has multiple risk factors, including smoking, genetic predisposition, environmental conditions, and dietary habits (2, 7). It is noteworthy that COPD is strongly associated with chronic inflammation, which may deteriorate during acute exacerbations, thereby posing a substantial threat to patients’ health (8). Hence, identifying effective methods to counteract abnormal inflammatory mechanisms in the lungs is pivotal for the prevention and early treatment of COPD.

In recent years, scholars have increasingly devoted attention to examining the correlation between dietary habits and COPD (9, 10). Specifically, the consumption of specific vitamins is believed to positively impact the maintenance of respiratory function and the alleviation of COPD symptoms. Owing to their antioxidant properties, these vitamins have the potential to facilitate cell repair and reduce inflammation, thereby potentially playing a pivotal role in both the prevention and management of COPD.

Niacin, alternatively referred to as vitamin B3, serves as a crucial nutrient participating in diverse physiological processes, such as energy metabolism, DNA repair, and cell signaling (11). Insufficient dietary intake of niacin may negatively impact oxidative phosphorylation, subsequently compromising mitochondrial respiratory function (12). However, controversy exists concerning niacin’s effects on human health. On one hand, studies indicated that niacin significantly decreases coronary events and overall mortality (13, 14). Conversely, certain research suggested that niacin may not be effective in preventing cardiovascular diseases and could potentially increase all-cause mortality (15–17). Furthermore, high dietary niacin intake has been positively associated with diabetes in US adults (18). Nevertheless, another study found that increasing dietary niacin intake improves glucose homeostasis in adults over 40 years old (19). Dietary niacin intake has also been linked to reduce all-cause and cardiac-related mortality in patients with chronic kidney disease (CKD) (20). Moreover, have revealed a notable correlation between niacin intake and forced expiratory volume in 1 s (FEV1) (21). This finding indicates a possible role of niacin in improving lung function. Another study has uncovered an inverse relationship between dietary niacin intake and the incidence of COPD among adults in the United State (22). A comparable correlation has been noted in the Korean populace as well (23). Nevertheless, the precise connection between dietary niacin intake and mortality in individuals with COPD remains obscure.

We hypothesize that there is a potential association between dietary niacin intake and COPD mortality. To test this hypothesis, we conducted a cohort study. The aim of this study is to explore the potential relationship between dietary niacin intake and mortality in COPD patients, providing scientific evidence to support nutritional interventions and treatments for COPD patients.

## Methods

### Data sources

This population-based longitudinal study has analyzed publicly available data from the NHANES database spanning from 1999 to 2018. Data were collected through structured interviews conducted in participants’ homes, physical examinations and laboratory tests performed at mobile examination centers, utilizing a multistage probability sampling design. All NHANES protocols were approved by the Ethical Review Board of the National Center for Health Statistics at the CDC, with written informed consent obtained from all survey participants (24). Since this study was based on anonymized publicly available deidentified data and informed consent was waived, ethical approval and consent were not required, so the Ethics Review Committee of the Second People’s Hospital of Banan District exempted the study.

**Figure 1:**
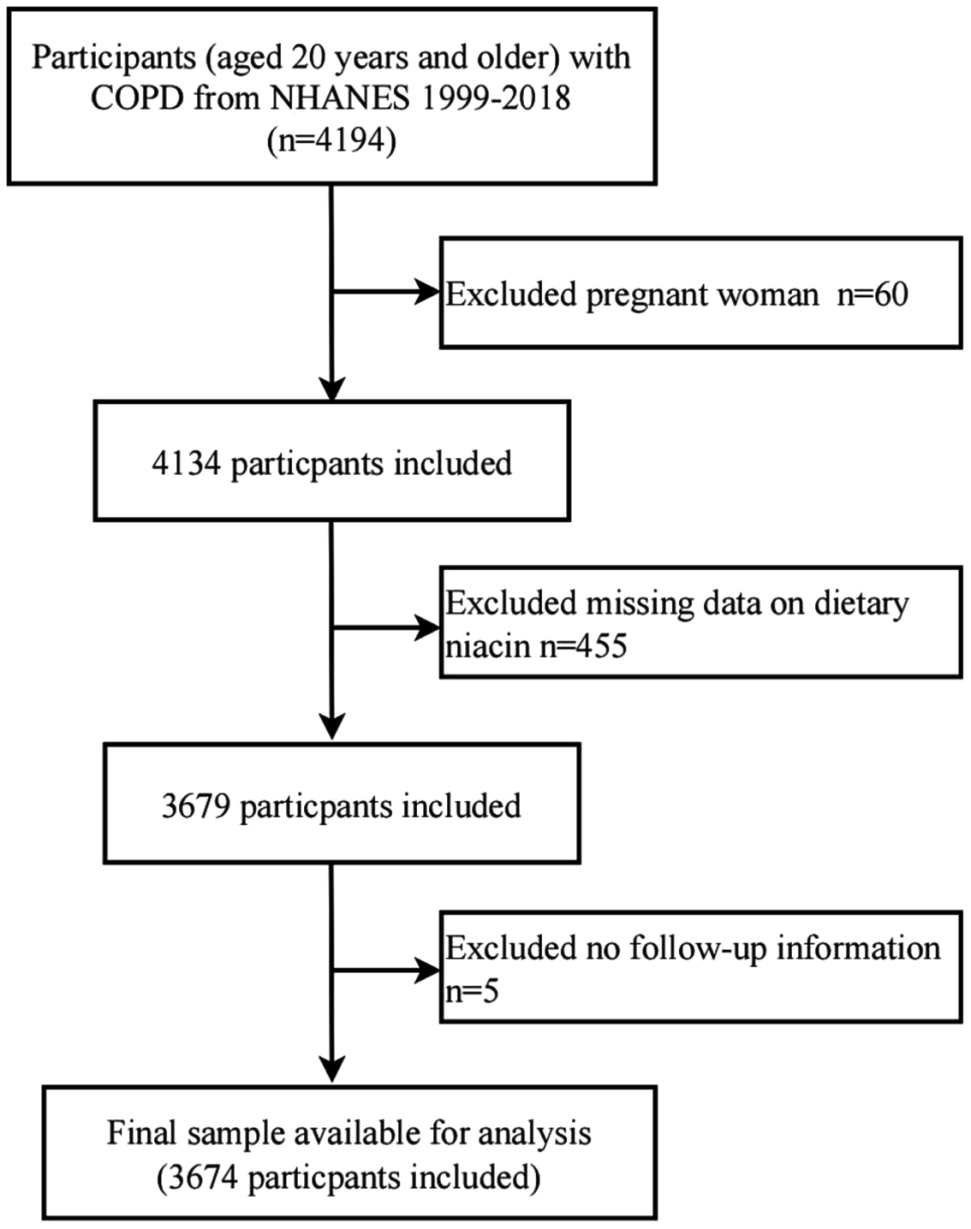
Flow diagram of the screening and enrollment of study participants.

For this study, 10 NHANES cycles from 1999 to 2018 were selected for further analysis. The presence of COPD was determined based on responses to the health questionnaire regarding disease status (25–27). Participants completed three self-administered questionnaires to verify their disease status: “Has a doctor or other health professional ever told you that you have chronic bronchitis?,” “Has a doctor or other health professional ever told you that you have emphysema?,” and “Has a doctor or other health professional ever told you that you have COPD?.” Participants who answered “yes” to any of these questions were placed in the COPD group (*n* = 4,194). After excluding participants who self-reported as pregnant (*n* = 60), those with missing dietary intake information (*n* = 455), and those without follow-up data (*n* = 5), a total of 3,674 COPD participants were included in the final analysis (Figure 1).

### Exposure variables

In the NHANES dietary survey, participants reported their food consumption over a 24-h period, specifying both the items consumed and their quantities. Dietary intake data from 1999 to 2001 was collected using the NHANES Computer-Assisted Dietary Interview (CADI) system. From 2002 onwards, the United States Department of Agriculture began employing the Automated Multiple-Pass Method (AMPM) to gather dietary consumption data. This fully computerized recall system comprehensively incorporates standardized questions and potential responses specific to various food items. Utilizing both CADI and AMPM, precise nutritional values were calculated based on each individual’s consumption of foods and beverages (28). With the exception of participants from the 1999–2000 and 2001–2002 cycles, all participants underwent two dietary recall interviews: an initial face-to-face session, followed by a telephone interview conducted within 3 to 10 days. Dietary niacin intake was derived from 24-h recall data, using an average value for participants with 2 days of data, following previous methods, and a single value for the minority of participants with 1 day of data (29). Following which, participants were categorized into tertiles based on their dietary niacin intake.

### Outcome ascertainment

All-cause mortality was determined using records from the National Death Index (NDI) up until December 31, 2019, and these records were linked to the NHANES dataset. Cause-specific mortality was identified using the International Classification of Diseases, 10th Revision (ICD-10) codes. All-cause mortality, defined as death from any cause, was the primary endpoint of this investigation. Cardiovascular disease (CVD) mortality was defined using ICD-10 codes I00-I09, I11, I13, I20-I51, and I60-I69 (30).

### Covariates

The standardized questionnaire obtained information regarding sex, age, race/ethnicity, marital status, education level, family income, physical activity, smoking status, drinking status, body mass index (BMI), energy consumption, dietary supplement usage, and the presence of hypertension and diabetes. Educational levels were classified into three categories: < 9 years, 9–12 years, and ≥ 12 years of schooling. In line with the recommendations of a US government report, family income was stratified into three groups based on the Poverty Income Ratio (PIR): low (≤ 1.3), middle (> 1.3 to ≤ 3.5), and high (> 3.5) (31). Individuals who have never engaged in smoking behavior are categorized as those who have smoked fewer than 100 cigarettes in their lifetime. Among those who have smoked more than 100 cigarettes, a distinction is made based on their present smoking practices: those who are still smoking are considered to be currently engaging in smoking behavior, whereas those who have smoked more than 100 cigarettes in the past but have quit are identified as having previously engaged in smoking behavior. Participants who reported consuming at least 12 alcoholic beverages of any kind within a year were categorized as individuals who regularly consume alcohol. Physical activity was classified as sedentary (no leisure-time physical activity), moderate (at least 10 min of activity in the past 30 days that caused only light sweating or a slight to moderate increase in breathing or heart rate), or vigorous (at least 10 min of activity in the past 30 days that caused heavy sweating or an increase in breathing or heart rate). BMI was calculated using the standard formula: weight (kg) divided by the square of height (m). Dietary supplement use was determined based on questions about consumption of nutritional supplements and medications in the past month. In the dietary intake data section, energy intake was determined using data collected via two 24-h dietary recalls. The history of hypertension or diabetes was self-reported by participants, with confirmation relying on diagnoses made by medical professionals.

### Statistical analysis

A secondary analysis was conducted on publicly available datasets. Categorical variables were represented as proportions (%) and analyzed using Chi-square tests. For continuous variables exhibiting a normal distribution, the mean ± standard deviation was employed for representation, and One-Way ANOVA tests were conducted for statistical analysis. In cases where continuous variables did not follow a normal distribution, the median and interquartile range were utilized for descriptive purposes, and the Kruskal-Wallis test was applied for comparative analysis.

To maximize statistical efficiency and minimize bias, multiple imputations were performed for missing data. For the visualization of survival analysis, Kaplan–Meier curves were generated, and differences between survival curves were tested using the log-rank test. The Cox proportional hazards model was employed to assess the hazard ratios (HRs) and 95% confidence intervals (CIs) for the association between dietary niacin intake and all-cause and CVD mortality. In this investigation, a series of three models were progressively constructed to account for various confounding factors: Model 1 served as the baseline unadjusted model; Model 2 introduced adjustments for age, sex, and race/ethnicity; Model 3 further controlled for marital status, education level, PIR, physical activity, smoking status, drinking status, BMI, energy consumption, dietary supplements taken, hypertension, and diabetes. The linear trend was performed by entering the median value of each tertiles as a continuous variable in the models.

Additionally, subgroup analyses and interaction analyses were conducted to explore the relationship between dietary niacin intake and all-cause and CVD mortality, aiming to determine the consistency of results across various subgroups. The subgroups were defined based on sex (male, female), age (< 60 years, ≥ 60 years), education level (≤ 12 years, > 12 years), PIR (low, medium or high), and BMI (< 25 kg/m^2^, ≥ 25 kg/m^2^).

Several sensitivity analyses were performed to assess the durability of the study’s findings. Initially, to mitigate the risk of reverse causality bias, we excluded participants who passed away within the initial 2 years of follow-up (*n* = 229), leaving 3,445 subjects for analysis. Secondly, all missing covariate data were directly deleted to ensure data integrity and accuracy (*n* = 517). Lastly, as a supplementary investigation, the correlation between dietary niacin intake, encompassing intake from supplements, and both all-cause and CVD mortality was explored. Due to the absence of detailed dietary supplement intake information in cycles from 1999 to 2006, data from these cycles were excluded, focusing solely on data from 2007 to 2018 which encompassed dietary supplement intake details. Subsequently, calculations determined the total niacin intake, inclusive of both dietary and supplement sources.

All statistical analyses were performed using R Statistical Software (Version 4.2.2, The R Foundation)^1^ and Free Statistics analysis platform (Version 1.9, Beijing, China).^2^ Statistical significance was defined as a two-sided *p* value < 0.05.

## Results

### Characteristics of the participants

A total of 3,674 individuals were enrolled in the study, with a mean age of 57.9 ± 16.6 years. Of these participants, 42.3% were male, and 57.7% were female. The participants were categorized into tertiles based on their dietary niacin intake: T1 (< 16.2 mg/day), T2 (16.2–24.3 mg/day), and T3 (> 24.3 mg/day). Table 1 presents the baseline characteristics stratified by tertiles of dietary niacin intake. Participants with higher dietary niacin intake tended to be younger, more frequently male, Non-Hispanic White, married or cohabiting with a partner. They often exhibited lower levels of physical activity, a higher likelihood of smoking and alcohol consumption, and tended to have higher household incomes and educational attainment. Moreover, they appeared to have a reduced prevalence of diabetes and hypertension, and reported more frequent use of dietary supplements.

**Table 1:** Population characteristics by categories of dietary niacin intake.

Characteristics	Dietary niacin intake, mg/day^a^				
	Total	T1	T2	T3	*p* value
		(< 16.2)	(16.2–24.3)	(> 24.3)	
No.	3,674	1,225	1,224	1,225	
Age(year)	57.9 ± 16.6	59.8 ± 16.4	59.2 ± 15.9	54.7 ± 17.0	< 0.001
Sex, n (%)					< 0.001
Male	1,555 (42.3)	324 (26.4)	483 (39.5)	748 (61.1)	
Female	2,119 (57.7)	901 (73.6)	741 (60.5)	477 (38.9)	
Race/ethnicity, n (%)					< 0.001
Non-Hispanic White	2,252 (61.3)	708 (57.8)	795 (65)	749 (61.1)	
Non-Hispanic Black	670 (18.2)	217 (17.7)	222 (18.1)	231 (18.9)	
Mexican American	302 (8.2)	138 (11.3)	79 (6.5)	85 (6.9)	
Others	450 (12.2)	162 (13.2)	128 (10.5)	160 (13.1)	
Education Level (year), n (%)					< 0.001
< 9	440 (12.0)	209 (17.1)	140 (11.4)	91 (7.4)	
9–12	1,622 (44.1)	564 (46)	529 (43.2)	529 (43.2)	
>12	1,612 (43.9)	452 (36.9)	555 (45.3)	605 (49.4)	
Marital Status, n (%)					< 0.001
Married or living with a partner	1906 (51.9)	577 (47.1)	645 (52.7)	684 (55.8)	
Living alone	1768 (48.1)	648 (52.9)	579 (47.3)	541 (44.2)	
Body mass index (kg/m^2^)	30.5 ± 8.1	30.4 ± 7.8	30.8 ± 8.3	30.2 ± 8.2	0.267
Family income, n (%)					< 0.001
Low	1,467 (39.9)	561 (45.8)	488 (39.9)	418 (34.1)	
Medium	1,476 (40.2)	462 (37.7)	495 (40.4)	519 (42.4)	
High	731 (19.9)	202 (16.5)	241 (19.7)	288 (23.5)	
Physical activity, n (%)					< 0.001
Sedentary	2072 (56.4)	746 (60.9)	723 (59.1)	603 (49.2)	
Moderate	893 (24.3)	282 (23)	298 (24.3)	313 (25.6)	
Vigorous	709 (19.3)	197 (16.1)	203 (16.6)	309 (25.2)	
Smoking status, n (%)					0.102
Never	1,110 (30.2)	405 (33.1)	359 (29.3)	346 (28.2)	
Former	1,306 (35.5)	411 (33.6)	444 (36.3)	451 (36.8)	
Current	1,258 (34.2)	409 (33.4)	421 (34.4)	428 (34.9)	
Drinking status, n (%)					< 0.001
No	947 (25.8)	386 (31.5)	341 (27.9)	220 (18)	
Yes	2,727 (74.2)	839 (68.5)	883 (72.1)	1,005 (82)	
Hypertension, n (%)					0.004
No	1962 (53.4)	613 (50)	653 (53.3)	696 (56.8)	
Yes	1712 (46.6)	612 (50)	571 (46.7)	529 (43.2)	
Diabetes, n (%)					0.04
No	2,881 (78.4)	957 (78.1)	936 (76.5)	988 (80.7)	
Yes	793 (21.6)	268 (21.9)	288 (23.5)	237 (19.3)	
Dietary supplements taken, n (%)					0.093
No	1974 (53.7)	689 (56.2)	639 (52.2)	646 (52.7)	
Yes	1700 (46.3)	536 (43.8)	585 (47.8)	579 (47.3)	
Energy consumption (kcal/d)	1787 (1,350, 2,349)	1,289 (1,010, 1,616)	1796 (1,460, 2,160)	2,457 (1976, 3,069)	< 0.001

a T1–T3, tertiles based on dietary niacin intake.

### Association of dietary niacin intake with all-cause and CVD mortality

**Figure 2:**
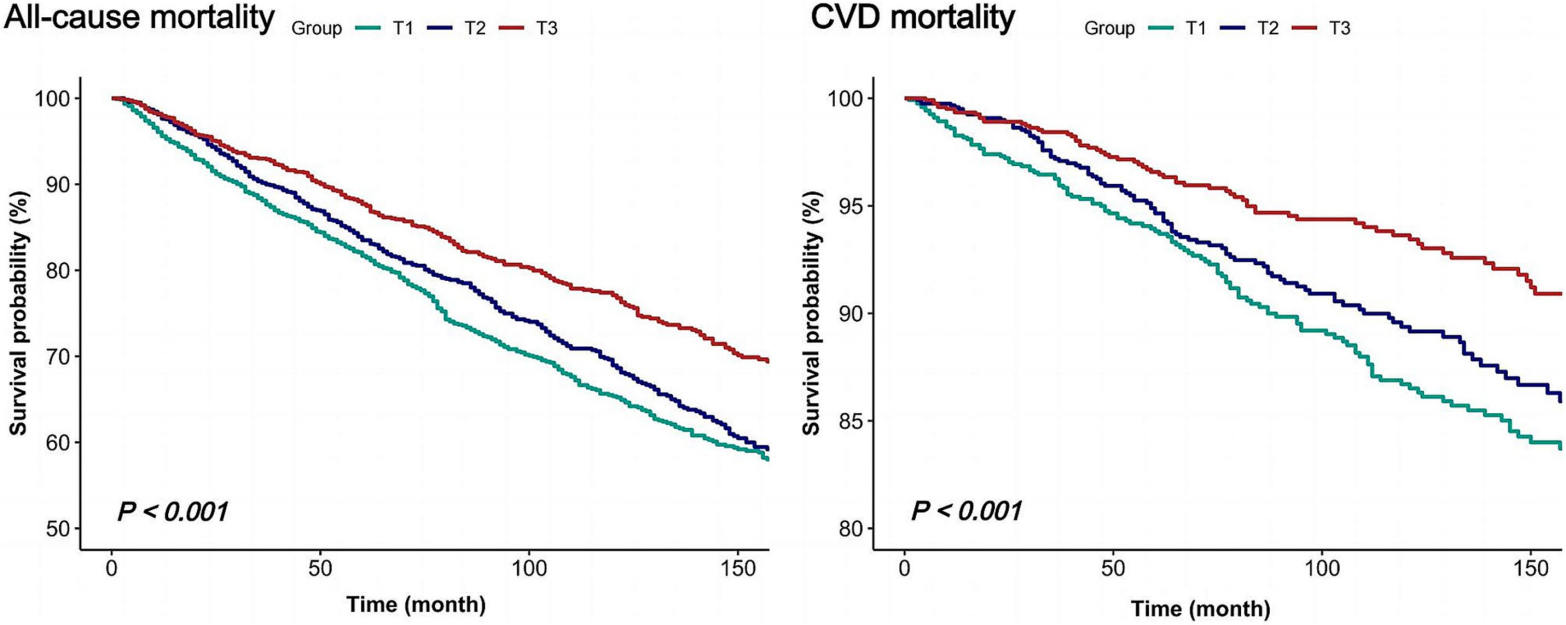
Kaplan–Meier survival curves for all-cause and CVD mortality of COPD individuals.

During an average follow-up of 8.3 years, a total of 1,085 all-cause deaths were observed, including 323 CVD-related deaths. To investigate the independent impact of dietary niacin intake on all-cause and CVD mortality among individuals with COPD, this study constructed three models. Multivariate adjustments included sex, age, race/ethnicity, marital status, PIR, education level, physical activity, smoking status, drinking status, BMI, energy consumption, dietary supplement use, hypertension and diabetes. After multivariable adjustment, higher dietary niacin intake was associated with reduced mortality risks in COPD patients (Table 2). Considering niacin intake as a continuous variable, each 10 mg/day increment correlated with a 12% decrease in all-cause mortality risk (HR: 0.88, 95% CI: 0.81–0.96, *p* = 0.004) and a 13% decrease in CVD mortality risk (HR: 0.87, 95% CI: 0.74–1.02, *p* = 0.091). Compared to the lowest tertile of niacin intake (reference HR 1.00), the second and third tertiles showed HRs of 0.79 (95% CI: 0.68–0.92, *p* = 0.003) and 0.67 (95% CI: 0.56–0.82, *p* < 0.001) for all-cause mortality, respectively (*p* for trend < 0.001). For CVD mortality, the corresponding HRs were 0.72 (95% CI: 0.55–0.95, *p* = 0.019) and 0.53 (95% CI: 0.37–0.77, *p* = 0.001; *p* for trend = 0.001). Kaplan–Meier survival curve analysis further confirmed the association, demonstrating lower all-cause and CVD mortality rates among participants in the highest tertile of dietary niacin intake compared to those in the lowest tertile (*p* < 0.001; Figure 2).

**Table 2:** Dietary niacin intake association with all-cause and CVD mortality among individuals with COPD.

Dietary niacin intake (mg/day)	No.	Event (%)	HR (95% CI)					
			Model 1^a^	*p* value	Model 2^b^	*p* value	Model 3^c^	*p* value
All-cause mortality								
Continuous per 10 mg/d increase	3,674	1,085 (29.5)	0.86 (0.81–0.91)	<0.001	0.9 (0.84–0.96)	0.001	0.88 (0.81–0.96)	0.004
T1 (< 16.2)	1,225	427 (34.9)	1(Reference)		1(Reference)		1(Reference)	
T2 (16.2–24.3)	1,224	372 (30.4)	0.90 (0.79–1.04)	0.15	0.82 (0.71–0.95)	0.007	0.79 (0.68–0.92)	0.003
T3 (> 24.3)	1,225	286 (23.3)	0.66 (0.57–0.77)	<0.001	0.71 (0.60–0.83)	<0.001	0.67 (0.56–0.82)	<0.001
*p* for trend				<0.001		<0.001		<0.001
CVD mortality								
Continuous per 10 mg/d increase	3,674	323 (8.8)	0.83 (0.74–0.92)	0.001	0.88 (0.77–0.99)	0.038	0.87 (0.74–1.02)	0.091
T1 (< 16.2)	1,225	136 (11.1)	1(Reference)		1(Reference)		1(Reference)	
T2 (16.2–24.3)	1,224	112 (9.2)	0.85 (0.66–1.09)	0.203	0.76 (0.59–0.98)	0.037	0.72 (0.55–0.95)	0.019
T3 (> 24.3)	1,225	75 (6.1)	0.54 (0.41–0.72)	<0.001	0.59 (0.44–0.79)	<0.001	0.53 (0.37–0.77)	0.001
*p* for trend				<0.001		<0.001		0.001

a Model 1: No adjusted.

b Model 2: Age, sex, race/ethnicity.

c Model 3: Model 2 + marital status, education level, family income, physical activity, smoking status, drinking status, body mass index, energy consumption, dietary supplements taken, hypertension and diabetes.

### Subgroup analyses and sensitivity analyses

**Figure 3:**
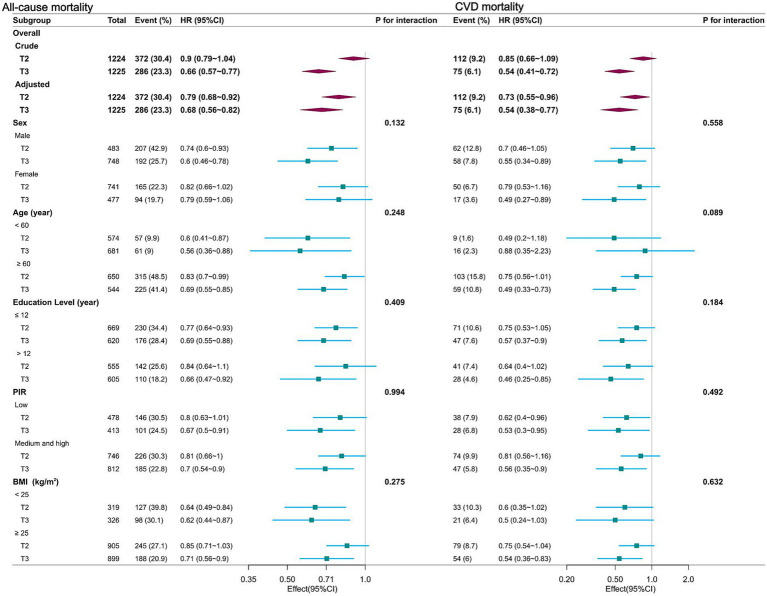
Forest plot of multivariable logistics analysis between dietary niacin intake and mortality among patients with COPD. Each square represents the HR with its 95% CI for a specific comparison or subgroup. The top of the plot represents the overall summary estimate. Adjusted for age, sex, race/ethnicity, marital status, education level, family income, physical activity, smoking status, drinking status, body mass index, energy consumption, dietary supplements taken, hypertension and diabetes. Note: dietary niacin intake tertiles (mg/day): T1 (reference, < 16.2); T2 (16.2–24.3); T3 (> 24.3).

Stratified analyses, categorized by sex, age, education level, PIR, and BMI, consistently demonstrated an association between dietary niacin intake and both all-cause and CVD mortality (Figure 3). There were no significant interactions (*p* for interaction > 0.05).

Sensitivity analyses excluding individuals who died within the first 2 years of follow-up revealed that the inverse associations between dietary niacin intake and both all-cause and CVD mortality remained essentially unchanged (Supplementary Table S1). These findings held true even after excluding observations with missing covariates (Supplementary Table S2). After incorporating dietary niacin supplement intake, a significant inverse association was found between total niacin intake (from both food and supplements) and all-cause and CVD mortality (Supplementary Table S3).

## Discussion

To the best of current knowledge, this is the first prospective study exploring the relationship between dietary niacin intake and all-cause and CVD mortality among individuals with COPD. The study reveals that higher dietary niacin intake is associated with a reduced risk of all-cause and CVD mortality. This association is independent of traditional risk factors, including dietary and lifestyle factors, as well as comorbidities. Various stratified analyses and sensitivity analyses demonstrate the robustness of the results.

Niacin possesses various crucial biological functions, among which its antioxidant properties are particularly significant for human health (32). However, the association between niacin and mortality risk has been inconsistent across different populations. Previous studies have found that niacin can significantly reduce coronary events and overall mortality (13, 14, 33). Nevertheless, some research has also suggested that niacin therapy offers limited benefits in preventing cardiovascular events (15, 16), and sole supplementation with niacin may increase all-cause mortality (17, 34). In a prospective cohort of 660 stable renal transplant recipients, a lower niacin status was associated with a higher risk of all-cause premature death (35). An American study involving 3,504 cancer patients indicated a negative correlation between niacin intake and mortality outcomes among cancer patients (36). This correlation persisted across various subgroups, including sex, age, and BMI. Another cohort study, encompassing 4,315 participants with nonalcoholic fatty liver disease (NAFLD), revealed that higher dietary niacin intake might be associated with a lower risk of all-cause mortality in NAFLD patients, although no significant negative correlation was observed with CVD mortality (37).

In the field of COPD research, the role of niacin has garnered attention. A nutritional epidemiological study involving 2,337 American smokers found a significant association between niacin and FEV1, suggesting that niacin may contribute to improving lung function (21). Another study, encompassing 7,055 American adults, revealed a negative correlation between dietary niacin intake and the incidence of chronic obstructive pulmonary disease (22). Similar conclusions were drawn by Shi, Yushan et al. in a study of 7,170 middle-aged and elderly individuals (38). A cross-sectional study conducted in Korea, involving 22,948 participants, discovered a significant association between niacin intake and reduced COPD severity among elderly men (aged ≥ 60) (23). Furthermore, a study of 7,615 Korean women indicated a negative correlation between lower niacin content in refined diets and predicted forced vital capacity levels (FVC) and FEV1 (39). These findings suggest that niacin may have some positive impact on lung function.

Patients with COPD not only suffer from declined lung function but also frequently suffer from systemic inflammation and vascular dysfunction (5), which accelerate atherosclerosis and increase the risk of cardiovascular events such as myocardial infarction and stroke (6). Smoking habits and low oxygen levels in COPD patients impose an additional burden on the heart, potentially promoting heart failure (40). Studies indicate that some COPD patients exhibit altered cardiac repolarization, potentially increasing the risk of arrhythmias and sudden cardiac death (41). Additionally, acute exacerbations of COPD (AECOPD) are a major cause of hospitalization and mortality in these patients (42).

Through a large-scale, long-term follow-up multi-ethnic cohort study, we found a negative correlation between dietary niacin intake and all-cause and CVD mortality among COPD patients. This association may be attributed to the various biological activities of niacin. The lungs of COPD patients suffer from chronic inflammation and oxidative stress damage over the long term, which are significant factors contributing to decreased lung function and disease progression (43, 44). Niacin, as an effective antioxidant, plays a crucial role in maintaining mitochondrial function (32). A lack of niacin in the diet may affect the oxidative phosphorylation process, thereby disrupting mitochondrial respiration (11). Animal experiments have shown that high doses of niacin significantly reduce lung inflammation, decrease the production of proinflammatory cytokines, and alleviate lung tissue damage by regulating the NF-κB signaling pathway (45). Furthermore, niacin plays a crucial role in regulating the production and release of inflammatory mediators. Niacin may modulate the production and release of inflammatory mediators, reducing lung inflammation and improving respiratory function and overall health status (46). In COPD patients, systemic and pulmonary inflammation are significant contributors to disease progression and increased CVD risk. Through its anti-inflammatory effects, niacin may help alleviate pulmonary inflammatory responses and decrease systemic inflammation levels, thereby reducing the risk of CVD. Additionally, niacin regulates the function of vascular endothelial cells, promoting the synthesis and release of nitric oxide, which improves endothelial function and mitigates vascular aging (47). Vascular dysfunction is a key factor that accelerates the development of atherosclerosis in COPD patients (48). By enhancing vascular endothelial function, niacin may help reduce vascular damage and lower the risk of CVD. As nicotinamide adenine dinucleotide (NAD), niacin participates in various adenosine diphosphate (ADP)-ribosylation reactions, influencing immune system stability (49). Immune dysfunction is a major contributor to disease progression and increased CVD risk in COPD patients (50). Moreover, chronic inflammation and oxidative stress can lead to DNA damage in COPD patients (51). In this regard, NAD plays a key role in the DNA repair mechanism. Substantial evidence suggests that NAD is involved in the repair of DNA damage through the base excision repair pathway, which is crucial for maintaining genomic stability and normal cellular function (52). This reduces cellular apoptosis and necrosis, lowering the risk of CVD. However, further research is needed to confirm these findings and explore the potential mechanisms underlying niacin’s beneficial effects in COPD patients.

This long-term follow-up prospective cohort study offers significant advantages. Primarily, it is the first to evaluate the relationship between dietary niacin intake and both all-cause and CVD mortality in a sample of adult individuals with COPD in the United States. Furthermore, the meticulous adjustment for various potential confounding factors, such as diet, lifestyle, and comorbidities, ensures the reliability and validity of the findings. However, the study also has its limitations. Firstly, due to its observational nature, it cannot definitively establish a causal link between dietary niacin intake and mortality rates; it can only identify associations. Secondly, the use of 24-h dietary recall questionnaires as the primary assessment tool may not fully capture the dynamic fluctuations in niacin intake over time, thereby limiting the ability to assess time-varying associations. Additionally, the NHANES database lacked consistent and comprehensive information on breastfeeding status among female participants. Given that the recommended dietary allowance for niacin differs between breastfeeding and non-breastfeeding women, the failure to account for this could introduce bias into our results. Finally, and most importantly in the context of this study, the lack of detailed data on the severity of COPD is a substantial constraint. As the NHANES database did not provide FEV1 predicted% data, we were unable to stratify our analysis according to COPD severity stages as recommended by the GOLD guidelines. This lack of stratification restricts a deeper understanding of the potential differential effects of dietary niacin intake on mortality across different stages of COPD progression. It also limits our ability to draw more precise conclusions about the role of niacin in the context of varying disease severities. Future research is strongly encouraged to address these limitations. Specifically, studies should aim to collect more comprehensive and longitudinal dietary data to better capture the time-varying nature of niacin intake. Moreover, future investigations should prioritize the inclusion of detailed COPD severity data, such as FEV1 predicted%, to enable a more nuanced analysis of the relationship between dietary niacin intake and disease progression, as well as mortality. This will ultimately contribute to the formulation of more accurate and personalized nutritional guidelines for COPD patients.

## Conclusion

We observed that a higher dietary intake of niacin was associated with lower all-cause and CVD mortality among individuals with COPD. These findings suggest that optimizing niacin intake may have beneficial effects on COPD outcomes and patient safety. However, since our analysis did not account for COPD severity, these results should be interpreted cautiously as preliminary assumptions. Further studies incorporating disease severity assessment are required to confirm and expand upon these findings.

## Data Availability

The datasets presented in this study can be found in online repositories. The names of the repository/repositories and accession number(s) can be found at: https://www.cdc.gov/nchs/nhanes/.

## Footnotes

^1^ http://www.R-project.org

^2^ http://www.clinicalscientists.cn/freestatistics
