# Supplementary material for: Association between dietary niacin intake and mortality among US individuals with chronic obstructive pulmonary disease: data from the national health and nutrition examination survey 1999–2018
Source: Front Nutr. 2025 Jun 9;12:1471549. doi: 10.3389/fnut.2025.1471549 (PMC12183066; doi:10.3389/fnut.2025.1471549)
Supplement: Supplementary file 1 [file Table_1.doc]

**Supplementary Material for**

**Association between Dietary Niacin Intake and Mortality among U.S. Individuals with Chronic Obstructive Pulmonary Disease: Data from the National Health and Nutrition Examination Survey 1999–2018**

**Table S1.** HRs (95% CIs) of all-cause and CVD mortality according to dietary niacin intake among COPD after excluding participants who died within two years of follow-up in NHANES 1999-2018.

| **Dietary niacin intake** | **No.** | **Event (%)** | **HR (95% CI)** | | | | | |
| --- | --- | --- | --- | --- | --- | --- | --- | --- |
|  |  |  | **Model 1^a^** | ***P* value** | **Model 2^b^** | ***P* value** | **Model 3^c^** | ***P* value** |
| **All-cause mortality** | | | | | | | | |
| Continuous per 10 mg/d increase | 3445 | 856 (24.8) | 0.87 (0.81~0.92) | <0.001 | 0.92 (0.85~0.99) | 0.022 | 0.89 (0.82~0.98) | 0.019 |
| T1 (< 16.3) | 1148 | 334 (29.1) | 1(Reference) |  | 1(Reference) |  | 1(Reference) |  |
| T2 (16.3–24.5) | 1148 | 300 (26.1) | 0.97 (0.83~1.14) | 0.714 | 0.88 (0.75~1.03) | 0.11 | 0.84 (0.7~0.99) | 0.039 |
| T3 (> 24.5) | 1149 | 222 (19.3) | 0.68 (0.58~0.81) | <0.001 | 0.74 (0.62~0.89) | 0.001 | 0.69 (0.55~0.85) | 0.001 |
| *P* for trend |  |  |  | <0.001 |  | 0.001 |  | 0.001 |
| **CVD mortality** | | | | | | | | |
| Continuous per 10 mg/d increase | 3445 | 264 (7.7) | 0.87 (0.77~0.97) | 0.013 | 0.95 (0.83~1.08) | 0.42 | 0.95 (0.8~1.12) | 0.524 |
| T1 (< 16.3) | 1148 | 106 (9.2) | 1(Reference) |  | 1(Reference) |  | 1(Reference) |  |
| T2 (16.3–24.5) | 1148 | 97 (8.4) | 0.98 (0.74~1.29) | 0.892 | 0.89 (0.67~1.18) | 0.418 | 0.86 (0.63~1.16) | 0.316 |
| T3 (> 24.5) | 1149 | 61 (5.3) | 0.59 (0.43~0.81) | 0.001 | 0.68 (0.49~0.94) | 0.02 | 0.62 (0.41~0.92) | 0.017 |
| *P* for trend |  |  |  | 0.001 |  | 0.022 |  | 0.018 |

^a^Model 1: No adjusted.

^b^Model 2: Age, sex, race/ethnicity.

^c^Model 3: Model 2 + marital status, education level, family income, physical activity, smoking status, drinking status, body mass index, energy consumption, dietary supplements taken, hypertension and diabetes.

**Table S2.** HRs (95%CIs) of all-cause and CVD mortality according to dietary niacin intake among COPD after all missing covariates were deleted in NHANES 1999-2018.

| **Dietary niacin intake** | **No.** | **Event (%)** | **HR (95% CI)** | | | | | |
| --- | --- | --- | --- | --- | --- | --- | --- | --- |
|  |  |  | **Model 1^a^** | ***P* value** | **Model 2^b^** | ***P* value** | **Model 3^c^** | ***P* value** |
| **All cause mortality** | | | | | | | | |
| Continuous per 10 mg/d increase | 3157 | 897 (28.4) | 0.85 (0.8~0.91) | <0.001 | 0.88 (0.82~0.95) | 0.001 | 0.88 (0.8~0.97) | 0.008 |
| T1 (< 15.6) | 1052 | 349 (33.2) | 1(Ref) |  | 1(Ref) |  | 1(Ref) |  |
| T2 (15.6–24.5) | 1052 | 316 (30) | 0.96 (0.82~1.11) | 0.557 | 0.83 (0.71~0.98) | 0.023 | 0.83 (0.71~0.98) | 0.03 |
| T3 (> 24.5) | 1053 | 232 (22) | 0.67 (0.56~0.79) | <0.001 | 0.69 (0.58~0.83) | <0.001 | 0.69 (0.56~0.86) | 0.001 |
| *P* for trend |  |  |  | <0.001 |  | <0.001 |  | 0.001 |
| **CVD mortality** | | | | | | | | |
| Continuous per 10 mg/d increase | 3157 | 265 (8.4) | 0.84 (0.74~0.94) | 0.003 | 0.87 (0.76~1) | 0.044 | 0.87 (0.73~1.04) | 0.121 |
| T1 (< 15.6) | 1052 | 109 (10.4) | 1(Ref) |  | 1(Ref) |  | 1(Ref) |  |
| T2 (15.6–24.5) | 1052 | 95 (9) | 0.92 (0.69~1.21) | 0.529 | 0.77 (0.58~1.03) | 0.076 | 0.76 (0.56~1.03) | 0.074 |
| T3 (> 24.5) | 1053 | 61 (5.8) | 0.56 (0.41~0.76) | <0.001 | 0.58 (0.41~0.8) | 0.001 | 0.54 (0.36~0.8) | 0.002 |
| *P* for trend |  |  |  | <0.001 |  | 0.001 |  | 0.002 |

^a^Model 1: No adjusted.

^b^Model 2: Age, sex, race/ethnicity.

^c^Model 3: Model 2 + marital status, education level, family income, physical activity, smoking status, drinking status, body mass index, energy consumption, dietary supplements taken, hypertension and diabetes.

Table S3. HRs (95%CIs) of all-cause and CVD mortality according to dietary niacin supplement intake among COPD in NHANES 2007-2018.^a^

| **Total niacin intake** | **No.** | **Event (%)** | **HR (95% CI)** | | | | | |
| --- | --- | --- | --- | --- | --- | --- | --- | --- |
|  |  |  | **Model 1^b^** | ***P* value** | **Model 2^c^** | ***P* value** | **Model 3^d^** | ***P* value** |
| **All cause mortality** | | | | | | | | |
| Continuous per 10 mg/d increase | 2421 | 512 (21.1) | 0.94 (0.89~0.99) | 0.031 | 0.92 (0.86~0.98) | 0.007 | 0.95 (0.89~1) | 0.054 |
| T1 (< 17.9) | 807 | 215 (26.6) | 1(Ref) |  | 1(Ref) |  | 1(Ref) |  |
| T2 (17.9–27.7) | 807 | 154 (19.1) | 0.67 (0.55~0.83) | <0.001 | 0.64 (0.52~0.79) | <0.001 | 0.64 (0.51~0.8) | <0.001 |
| T3 (> 27.7) | 807 | 143 (17.7) | 0.67 (0.54~0.83) | <0.001 | 0.6 (0.49~0.75) | <0.001 | 0.65 (0.51~0.84) | 0.001 |
| *P* for trend |  |  |  | <0.001 |  | <0.001 |  | 0.001 |
| **CVD mortality** | | | | | | | | |
| Continuous per 10 mg/d increase | 2421 | 138 (5.7) | 0.84 (0.73~0.97) | 0.02 | 0.8 (0.68~0.93) | 0.004 | 0.83 (0.69~0.99) | 0.036 |
| T1 (< 17.9) | 807 | 65 (8.1) | 1(Ref) |  | 1(Ref) |  | 1(Ref) |  |
| T2 (17.9–27.7) | 807 | 38 (4.7) | 0.55 (0.37~0.82) | 0.003 | 0.51 (0.34~0.76) | 0.001 | 0.52 (0.33~0.8) | 0.003 |
| T3 (> 27.7) | 807 | 35 (4.3) | 0.55 (0.37~0.83) | 0.005 | 0.48 (0.31~0.73) | 0.001 | 0.51 (0.31~0.83) | 0.007 |
| *P* for trend |  |  |  | 0.002 |  | <0.001 |  | 0.005 |

^a^Only cycles from the National Health and Nutrition Examination Survey 2007–2018 included a dietary niacin supplement questionnaire. Among these cycles, 2,421 participants reported a dietary niacin supplement intake of ≥ 0.1 mg/day. The total niacin intake includes contributions from both dietary and supplemental sources.

^b^Crude model.

^c^Adjusted for age, sex, race/ethnicity.

^d^Adjusted for age, sex, race/ethnicity, marital status, education level, family income, physical activity, smoking status, drinking status, body mass index, energy consumption, dietary supplements taken, hypertension and diabetes.
